# Intensification to injectable therapy in type 2 diabetes: mixed methods study (protocol)

**DOI:** 10.1186/s12913-019-4112-3

**Published:** 2019-05-03

**Authors:** Simon de Lusignan, William Hinton, Emmanouela Konstantara, Neil Munro, Martin Whyte, Julie Mount, Michael Feher

**Affiliations:** 1 Department of Clinical and Experimental Medicine, University of Surrey, The Leggett Building, Daphne Jackson Rd, Guildford, GU2 7XP UK; 2 Research and Surveillance Centre (RSC), Royal College of Practitioners (RCGP), 30 Euston Square, London, NW1 2FN UK; 3 Real World Evidence, Eli Lilly and Company, Lilly House, Priestly Road, Basingstoke, Hampshire RG24 9NL UK

**Keywords:** Diabetes mellitus, type 2, Insulin, Glucagon-like peptide-1 receptor, Focus groups, Surveys and questionnaires, Medical record systems, computerized, Electronic health records, Patients, General practice, Qualitative research

## Abstract

**Background:**

In the UK, type 2 diabetes mellitus (T2D) is largely managed in primary care. Delay in the intensification to injectable therapy, a form of clinical inertia, is associated with worse glycaemic control. UK general practice is highly computerised, with care being recorded on computerised medical record systems; this allows for quantitative analysis of clinical care but not of the underpinning decision-making process. The aim of this study is to investigate perceptions of patients and clinicians in primary care on the initiation of injectable therapies in T2D, and the context within which those decisions are made.

**Methods:**

This is a mixed methods study, taking a “realist evaluation” approach. The qualitative components comprise focus groups, interviews, and video recordings of simulated surgeries; the quantitative analysis: an overview of participating practices, elements of the video recording, and an online survey. We will recruit primary care clinicians (general practitioners and nurses) and patients from a representative sample of practices within the Royal College of General Practitioners (RCGP) Research and Surveillance Centre (RSC) network. Participants will be patients with T2D, and primary care clinicians. Focus groups and semi-structured interviews will be recorded, transcribed *verbatim* and analysed using Framework Analysis. The simulated surgeries will include cases that might be escalated to injectable therapy. The consultation will be reviewed using the Calgary-Cambridge model to assess communication and determination of adherence to national prescribing guidelines. We will conduct multi-channel video recording including screen capture, clinician and patient facial expressions, wide angle view of the consultation, and the computerised medical record screen. This allows annotation and qualitative analysis of the video recordings, and statistical analyses for the quantitative data. We will also conduct an online survey of primary care clinicians’ attitudes to, and perceptions of, initiation of injectable therapies, which will be analysed using summary statistics.

**Discussion:**

Results aim to provide a detailed insight into the dynamic two-way decision-making process underpinning use of injectable therapy for T2D. The study will provide insights into clinical practice and enable the development of training, interventions and guidelines that may facilitate, where appropriate, the intensification to injectable therapy.

**Electronic supplementary material:**

The online version of this article (10.1186/s12913-019-4112-3) contains supplementary material, which is available to authorized users.

## Background

Type 2 diabetes (T2D) is a major problem for many health care systems [1–8]. The World Health Organisation (WHO) has estimated that there are approximately 422 million people in the world with T2D [1]. Following analyses of the Framingham study in the 1970s it became clear that T2D is a major risk factor for macrovascular disease (including myocardial infarction and stroke), and studies since have demonstrated that macrovascular risk increases with worsening glycaemic control [9, 10]. Diabetes is also associated with an increased risk of microvascular complications which includes diabetic retinopathy, neuropathy, and renal disease [11]. Several landmark trials have demonstrated that risk for both microvascular and macrovascular complications of diabetes can be reduced by improving blood glucose control [12–15].

National and international guidelines have provided targets for optimum glycaemic control, which have been established through observational evidence and clinical trials [16–22]. However, adequate glycaemic control is difficult to achieve for a significant proportion of people with T2D. In a large-scale European study, real-world diabetes care was compared against the glycaemic targets produced by the American Diabetes Association (ADA) and European Association for the Study of Diabetes (EASD), and found that only 53.6% of people with T2D achieved adequate glycaemic control, with considerable variation between countries [23].

In the UK, T2D is largely managed in primary care, reinforced by pay-for-performance (P4P) in general practitioners’ contracts [2, 3, 24] - a scheme called the Quality and Outcomes Framework (QOF). UK general practice consultations are recorded into computerised medical record (CMR) systems which actively flag whether a patient with T2D is at target. Rewards paid through the P4P system, QOF, are solely based on information recorded in CMR systems. These computerised systems record the diagnosis of T2D and link to pathology services allowing transfer of glycated haemoglobin (HbA1c) data to the individual’s medical records. CMR systems also record all the prescriptions issued in primary care. Additionally, most brands of CMR systems prompt the primary care clinician at every consultation if a person with T2D is not at their glycaemic target. Therefore, every time a person with T2D presents to their general practitioner (GP) or other primary care clinician, the clinician can readily tell if they are at target, and generally with one mouse click, whether they are receiving maximal oral therapy [25].

QOF remuneration targets for T2D include optimising the number of people below set glycaemic thresholds. CMR based interventions are known to generally improve care [25] and the introduction of these P4P targets appear to have improved glycaemic control and reduced inequalities in T2D management [26, 27]. However, it is difficult to disentangle these effects from other quality improvement initiatives.

One component of suboptimal management is delay in intensification of therapy. Delays in intensification occur at each stage of treatment: from diagnosis to first oral medication, to second and third oral medications, to the initiation of injectable therapies and escalation of injectable therapies once initiated [28]. These delays termed “therapeutic inertia” or, more commonly “clinical inertia” [29], are associated with impaired glycaemic control and concomitant complications including microvascular (e.g. retinopathy, chronic kidney disease) and macrovascular diseases (e.g. heart failure, stroke) [30]. Despite improved glycaemic control following the use of injectable therapy, drawbacks of insulin include weight gain, and severe hypoglycaemia and increased risk of death [31, 32], whilst glucagon-like peptide 1 receptor agonists (GLP-1 RAs) have been linked to gastrointestinal symptoms such as nausea, vomiting, and diarrhoea [33], which may deter people from using them. Other barriers to starting injectable therapy include the individual struggling to acknowledge that their diabetes has progressed, anxiety and fear of pain from injecting, the need to regularly test blood glucose levels, and the difficulty incorporating injecting during working hours [34–36].

Clinician, patient, and health service factors have been identified as contributing to clinical inertia [37–44]. However, an improved understanding of the specific patient, clinician, and health service factors that influence clinical inertia is urgently needed to facilitate improved glycaemic control and health outcomes in people with T2D.

## Aims and objectives

The aim of this study is to explore the perceptions of patients and clinicians regarding the initiation of injectable therapies: insulin and GLP-1 RAs, and to describe the context within which these decisions are made.

The primary objectives of this study are:

- To explore the attitudes and experiences of patients with T2D and primary care clinicians (GPs and nurses) of barriers and facilitators to initiating injectable therapies.
- To describe the context and processes in which injectable therapy initiation would take place; in particular how computerised prompts are managed.
- To describe any consensus or discrepancies within and between clinicians and patients about attitudes to intensification to injectable therapies.

## Methods

### Study design and setting

This study combines qualitative and quantitative methods, and should be regarded as a mixed methods study [45–47]. It will be run within the Royal College of General Practitioners (RCGP) Research and Surveillance Centre (RSC) sentinel network of general practices (35, 36). The qualitative methods comprise focus groups that will be carried out with a sample of practices. Interviews will be used if patients or clinicians are unable or uncomfortable with participating in focus groups. The subsequent quantitative component will include a comparison of initial themes on injectable therapy drawn from an earlier phase of the study, with other practices in the network via a survey to explore consensus. We will also carry out a video study to provide quantitative data on key prompts/triggers for the initiation of injectable therapy raised during a consultation with a simulated T2D patient.

We will recruit participants from volunteer practices within the RCGP RSC network, [48, 49]; our aim is to recruit practices that are representative of the network. The RCGP RSC data have been used extensively for diabetes research, and although there are limitations in case finding, recording of pathology results, and in recording of therapies, the network has good data quality and is largely nationally representative [48].

**Fig. 1:**
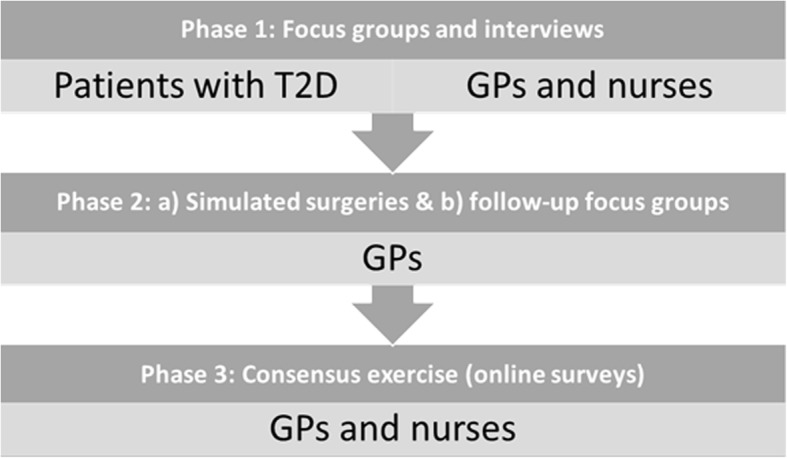
Overview of the study phases

The research project will be run in three phases: Phase 1: Focus groups and interviews; Phase 2: Video recording of simulated surgeries with follow-up focus groups; and Phase 3: Survey, consensus exercise (Fig. 1). The qualitative processes will follow the Consolidated Criteria for Reporting Qualitative Research (COREQ) checklist [50].

### Philosophical approach

We have defined, a priori, critical realism as an overall philosophical approach. This approach is appropriate because the decisions to intensify therapy in the clinical consultation are complex [51]. The Medical Research Council framework for Complex Intervention includes key elements of critical realism, in particular the need to define the context and mechanism [52].

#### Realist evaluation

We will use Pawson and Tilley’s “realist evaluation” to provide a lens through which to explore initiation of injectable therapy [53]. Realist evaluations explore how context interacts with a mechanism to produce an outcome. In this study:

- The context is the management of a person with T2D in primary care, where the primary care clinician should take a holistic view of the benefit-risk of any treatment option.
- The mechanism(s) are complex. There are evidence-based guidelines about intensification of treatment [54]; there are financial incentives to ensure that patients achieve glycaemic control [27]; and prompts that appear in the consultation [55].
- The outcome measure is the commencement of injectable therapy, in accordance with National Institute for Health and Care Excellence (NICE) guidance [54].

Critical realism attempts to answer the questions “What works, for whom, in what circumstances, in what respects, and why?” [56]. Our focus groups and direct observation of the clinical consultation (video of simulated surgery), and our consensus exercise will set out to explore these. This is in contrast to the traditional approach used in interventional studies (such as randomised controlled trials) which focus on whether an intervention works or not, without in-depth consideration of the context or analysis of the mechanisms [57].

### Theoretical framework – model of pathways to treatment

To help us interpret and understand the different pathways to initiating injectable therapy in people with T2D, we will use the Model of Pathways to Treatment as a theoretical framework [58]. This has previously been used to explore different care pathways in cancer research [59, 60], but has been little used in diabetes. The model pinpoints five key time points, “events”, that might occur before a health care professional starts treatment in an individual. These events include, (1) an individual identifying physical changes in the body; (2) realising that these symptoms are abnormal and should be discussed with a health care professional; (3) discussing concerns about the body changes with a health care professional for the first time; (4) the time point at which a diagnosis is made; and (5) commencement of treatment. The time periods, “intervals”, that occur between each event are factored into the model, as well as “processes”, which take place within the intervals and can trigger the successive event.

### Sample selection

Participants will be purposively sampled to ensure that a range of individual characteristics are represented within the staff (years in practice, diabetes experience) and patient (diabetes duration, ethnicity, socioeconomic status) focus groups [61]. Our sample will be restricted, for travelling reasons, to within 50 miles of our University location. Effectively, this is to London and the southeast of England. We will select practices that are broadly similar to other general practices in England in terms of size, prevalence of patients with T2D, their age, gender, ethnicity, deprivation, glycaemic control and threshold for prescribing injectable therapy. We report the sampling methods in more detail in the following sections.

### Data collection

#### Phase 1 – Focus groups and interviews

Phase 1 comprises separate focus groups, or interviews, with patients and clinicians. We will conduct separate focus groups for the different participating groups to minimise response bias: (1) patients, and (2) primary care clinicians.

Topics for the patient group will include:

(i) Views on initiating injectable therapy
(ii) Facilitators and barriers affecting initiation and adherence to injectable therapy
(iii) Other factors that affect decision-making.

Clinician topics will be:

(i) Reflection on their personal decision-making processes
(ii) Insights into barriers and facilitators to initiation and adherence to injectable therapy.

Whilst interaction within focus groups may identify themes that may not emerge in individual interviews [62], we may have to offer interviews when participants find focus groups daunting, or where there are difficulties with scheduling. The focus groups will comprise six to eight participants - the recommended number for this method [63].

We plan to conduct two patient focus groups and two practitioner focus groups. Within each study practice, we will recruit patients with T2D who are naïve to injectable therapy, and others who have prior experience of GLP-1 RAs and/or of insulin. The moderator will introduce the topics under discussion, monitor group dynamics to ensure that all participants have the opportunity to share their views, are adequately represented, and all topics are covered. A second moderator will be present to take detailed notes of the discussion and help the moderator keep to times. The focus groups will last between 90 to 120 min. The focus groups and interviews will be recorded and transcribed *verbatim* [50].

Pilot focus groups will be conducted to inform the interview schedules. One pilot focus group will be conducted with clinicians, and another with T2D patients.

##### Participant inclusion criteria

The following people will be eligible for phase 1 of the study:

- Aged 18 years or older
- A recorded and physician-confirmed diagnosis T2D
- English speaking
- A registered patient with one of the participating RCGP RSC practices.

Primary care clinicians will also be eligible for inclusion if they are a GP or a practice nurse working at one of the participating practices.

#### Phase 2 – a) Video-recorded simulated surgeries

We will conduct video-recorded simulated surgeries with GPs and follow-up focus groups. These will capture the context within which escalation to injectable therapy occurs. It will also provide direct observation of the components of the clinical consultation that are not routinely recorded in CMRs, such as clinicians’ approach towards care (shared or non-shared decision-making with patients). We hope to reproduce the prompts and access to guidelines that GPs generally see.

Six GPs and three actors will participate in a diabetes clinic run at their practices. Actors will be accessed through the RCGP Clinical Skills Assessment (CSA), as they have experience in simulated surgeries within a primary care environment, and through our contacts with patients with T2D. Concepts communicated by participants in Phase 1 will be used to inform scenarios involving initiation of injectable therapy (insulin and GLP-1 RAs), which will then be given to the actors.

The scenarios will include: (1) a simulated patient where injectable therapy is probably not needed (Additional file 1); (2) a simulated patient who should be initiated on insulin (Additional file 2); and (3) a simulated patient who should be initiated on a GLP-1 RA (Additional file 3).

The actors will present their scenario to each of the clinicians in rotation, which will be video-recorded using the Activity Log File Aggregation (ALFA) toolkit [64]. The booked duration of each consultation will be 20 min; they will be stopped at 22 min. Most general practice diabetes clinics allow 20 min for consultations, though some follow-up is done in 10 min.

The ALFA toolkit is a multi-channel video method to capture the precise observation of the clinical consultation such as verbal and non-verbal cues, and the impact of the computer, through capturing different streams of data [64, 65]. The ALFA toolkit records four streams of data; (1) clinician’s upper body, (2) patient’s upper body, (3) wider angle capturing both patient and clinician, and (4) the clinician’s computer screen. The videos will be stored on encrypted and password protected hard drives.

#### b) Follow-up focus groups – including assessing consensus statements

The GPs that take part in the simulated surgeries will be asked to participate in a follow-up focus group to discuss the scenarios that were presented to them. In addition, we will present the GPs with consensus statements to explore where there is a consensus or disagreement about the facilitators and barriers to intensification of treatment to injectable therapy [66]. These statements will be generated using the findings of the focus groups in phase 1. Such a process often assists to produce recommendations or issue statements that can be used for understanding the current state of care and assist in related policy development or process improvement [67–70]. The consensus statements will be subjected to an expert panel review (the GPs in this instance) during which they will indicate the appropriateness of each statement using a Likert scale similar to that used in Delphi studies [71]. The feedback will be used to revise the consensus statements and sent for a further round of consensus to the same expert panel.

##### Participant inclusion criteria

Up to six GPs working at one of the participating practices will participate in phase 2, and also three actors accessed via the RCGP CSA and through contact with patients with T2D.

#### Phase 3 – Quantitative surveys /consensus exercise

**Fig. 2:**
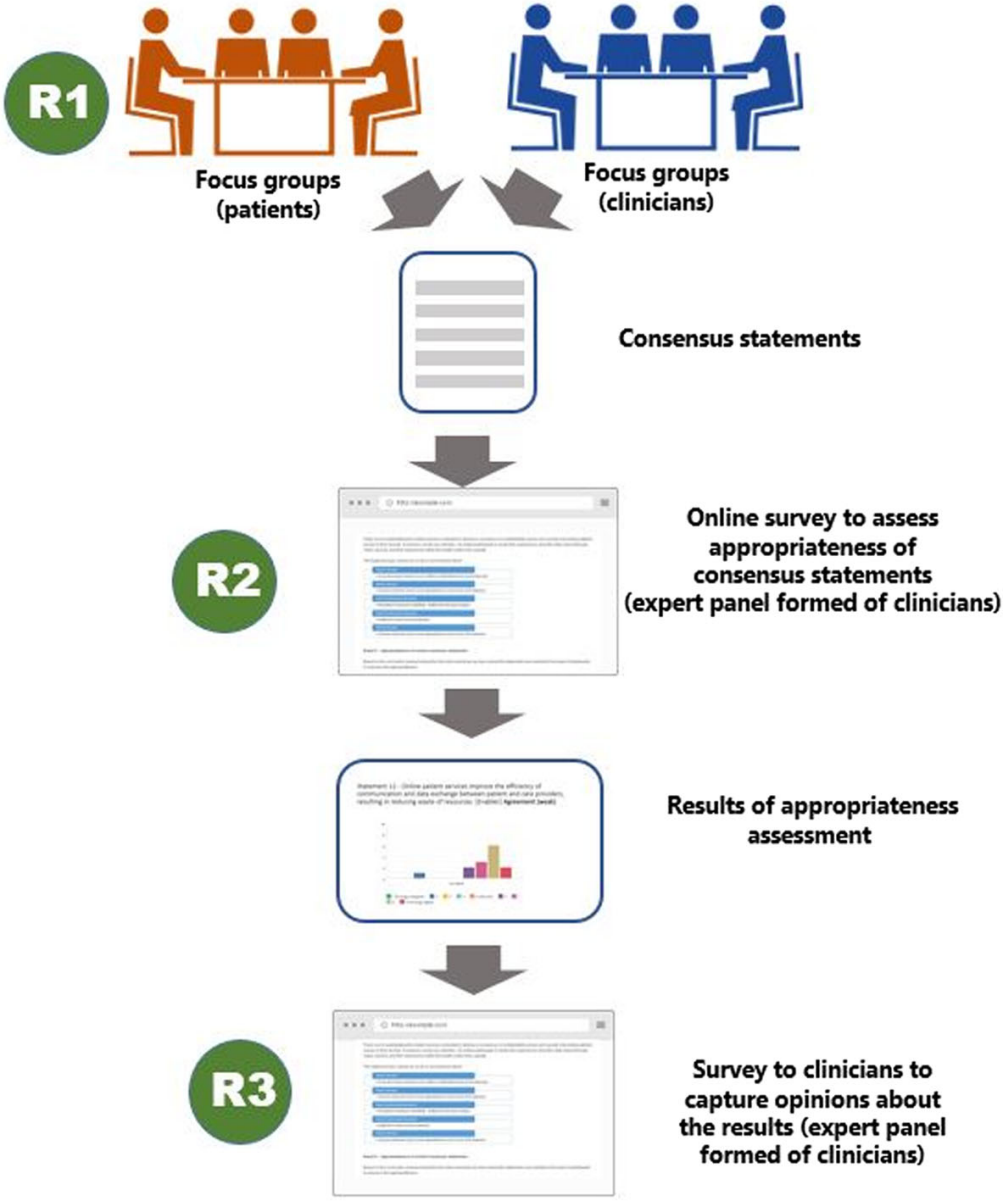
Consensus process pathway

We will use the findings from phases 1 and 2 (Round 1) to develop an online survey for clinicians, to capture the degree of consensus with our phase 1 and 2 findings (Round 2) (Fig. 2). The surveys will include statements for respondents to agree or disagree with. There will also be an opportunity for the respondents to explain why they agree or disagree with a statement.

- Generating consensus statements: This will be a selective round where the information captured in the research so far will be converted into a set of consensus statements (Round 2). The information captured from patients will be used to formulate a set of consensus statements for representing patient views, whilst information captured from the clinicians will be used to formulate a similar set of statements representing the views of clinicians. These consensus statements will be presented to the GPs during the follow-up focus groups in Phase 2. The clinicians will be expected to indicate the appropriateness of each of the statements using a 7 point Likert scale (Round 2).
- Looking for agreement, equivocation or disagreement with the consensus statements: During this final round the statements generated will be analysed and results will be presented to the clinicians as an online report (and survey to collect opinions about the results) (Round 3). This will be carried out in parallel to phase 3. For each of the statements, we will indicate if the panel of clinicians in round 1 were in agreement, disagreement or equivocal. During the presentations, the clinicians will be invited to express their opinion about the various levels of agreement assigned to the statements. In cases, where the majority of statements are not acceptable to the panel, we will consider conducting an additional round of consensus after revising the statements.
- This report will be shared with clinicians during the final round of the consensus process whereby the clinicians will be invited to suggest possible reasons for the any disagreements expressed during the evolution of the consensus document (Round 3).

##### Participant inclusion criteria

Experts that have a substantial amount of experience caring for people with T2D in the primary care setting (Round 2). We will also involve clinicians in the wider RCGP RSC network to explore whether the consensus feedback is relatable to their primary care setting (Round 3).

### Data analysis

#### Phase 1 – Focus groups and interviews

We will use Framework Analysis, exploring previously identified themes from existing literature (deductive analysis), as well as new concepts that might emerge during the data collection process (inductive analysis) [72].

This analysis involves a five-step process: (1) familiarisation; (2) identifying a thematic framework; (3) indexing; (4) charting; and (5) mapping and interpretation [73]. In the first step (1), the analyst will read the transcripts, reflect on the research question, and keep notes of potential ideas and recurring concepts. Then (2), the analyst will use a priori knowledge of the literature as well as the concepts from the first step to create a framework/index to sort the new material. This is expected to be a descriptive list of concepts initially, which will be refined to represent the diversity, centrality, and dynamics of participants’ attitudes. Once this framework has been developed, (3) it will be applied systematically to the data, using a numerical system that will link them directly to the index. Where a passage connects to more than one theme, this will be noted. At this stage, a second analyst will use the framework to test the transparency of the method and compare his assumptions with the first analyst’s. Once this is completed, the indexes will be used to create thematic charts (4), which will include a refined summary of major subjects that will have emerged. This will provide a more abstract view of the data in preparation of the last step (5), wherein the analyst will look and interpret the data as a whole.

This approach has been applied extensively in policy research [74, 75], to observe similarities, discrepancies, and interrelationships among the data [76]. We will use QSR NVivo 11 and Microsoft Excel software to organise and analyse this data [77].

#### Phase 2 – Simulated surgeries and follow-up focus groups

The ALFA toolkit [64] will be used to collect video-recorded data from the simulated surgeries. These will be analysed via thematic coding using the themes developed from the focus groups [78]. Since the ALFA toolkit captures several streams of data, we will analyse different aspects of the consultation: non-verbal communication of the patient and clinician, verbal communication, and information that is entered onto the computer.

##### Assessment of the consultation quality using the Calgary-Cambridge model

The Calgary-Cambridge consultation model [79] will be used to appraise the primary care clinicians’ consultation skills from the simulated surgeries. This enables us to assess if there has been shared understanding and decision-making [80–83]. The Calgary-Cambridge consultation model has five steps: (1) initiating the session; (2) gathering information; (3) building the relationship; (4) explanation and planning; and (5) closing the session. The Global Consultation Rating Scale, based on the Calgary-Cambridge will be used to assess the quality of communication [84].

##### Assessment of the interaction between the GP and the simulated patient

A checklist will be developed for each simulated surgery to enable us to:

1. Highlight key prompts/triggers for action either in the patient’s history or the simulated medical record
2. Note whether these were stated or accessed during the consultation;
3. Whether the prompts / triggers resulted in action;
4. To determine whether the outcome of the consultation which would be anticipated if guidelines were followed.

This will be carried out using expert reviewers. These reviewers will first identify key triggers in the records or history from the simulated patient which should trigger action, in this case intensification of T2D therapy. These will be identified by two expert reviewers, and any differences discussed. The videos will then be independently reviewed by two experts to see if these triggers were recognised, discussed, and actioned. This peer approach will mirror similar methods used to assess multidisciplinary team meetings [85].

#### Phase 3 – Survey/consensus exercise

Summary statistics will be used to describe the population of interest. We will report these as agreement, disagreement or equivocation where there is a spread of Likert scale responses for each statement.

### Data collection and analysis for consensus building

The consensus building process will be integrated with the focus group activities planned in the study. This will be carried out in parallel to the three phases described above.

### Integration of the data from each study phase

As described above, the first phase of the study will inform the design of the second and third phases of the study. The study will therefore, use a sequential exploratory design in which qualitative data will be collected and analysed in the first instance, followed by the collection and analysis of quantitative data, and these will then be integrated and interpreted [86].

## Discussion: strengths and weaknesses

One of the strengths of the study is its representativeness. The RCGP RSC sentinel network provides access to a nationally representative sample of real world evidence (RWE) data. This can facilitate the development of interventions that have real-world effectiveness [87].

What this study adds is a robust exploration from a realist evaluative stance. The study aims to capture the details of context and mechanism and how these combine to affect outcome (in this case commencing injectable therapy for diabetes).

Our context is one where T2D is largely managed by GPs and nurses in primary care. A registration based system where one patient is seen in a single practice, often by the same doctor or nurse for their diabetes care. These general practices are highly computerised, and the consultations will be recorded on the practice CMR system. The CMR also allows all previous blood tests including glycated haemoglobin to be readily visualised.

The mechanism for achieving change includes prompts and guidance pointing out poor glycaemic control. These reminders may nudge the clinician towards escalation of treatment. There is also the P4P/QOF incentive to intensify treatment and achieve glycaemic control and other indicator standards. These factors are in addition to those already described in the literature.

A weakness of the study is that as language is an important aspect of qualitative research, only English-speaking individuals can participate. This means that perceptions of people with different language may not be explored. Whilst video is much used for training and assessment in primary care, there are always concerns it may interfere with the clinician-patient relationship. However, research suggest that consultation behaviour is little affected by awareness of video recording [64].

In summary, we will explore the topic of intensification to injectable therapy using a triangulation of qualitative and quantitative research methods. The use of video recorded simulated surgeries to explore themes that were derived from focus groups is a novel approach, but we feel necessary to capture information about context and mechanise that may not emerge from the narrative. This study will gain insight into the different dimensions of highly context-dependent settings, in our case general practice.

## Additional files

Additional file 1: Scenario and medical record of Patient 1 (John Thompson). Scenario and medical record of Patient 1 (John Thompson) (DOCX 14 kb)

Additional file 2: Scenario and medical record of Patient 2 (Jane Smith). Scenario and medical record of Patient 2 (Jane Smith) (DOCX 14 kb)

Additional file 3: Scenario and medical record of Patient 3 (Gary Jones). Scenario and medical record of Patient 3 (Gary Jones) (DOCX 14 kb)

## Abbreviations

ADA: American Diabetes Association
ALFA: Activity Log File Aggregation
CMR: Computer medical record
COREQ: Consolidated criteria for reporting qualitative research
CSA: Clinical Skills Assessment
EASD: European Association for the Study of Diabetes
GLP-1 RAs: Glucagon-like peptide 1 receptor agonists
GPs: General Practitioners
HbA1c: Haemoglobin A1c (glycated haemoglobin)
NICE: National Institute for Health and Care Excellence
QOF: Quality and Outcomes Framework
RCGP RSC: Royal College of General Practitioners Research and Surveillance Centre
RWE: Real World Evidence
T2D: Type 2 Diabetes Mellitus
