# Supplementary material for: Intensification to injectable therapy in type 2 diabetes: mixed methods study (protocol)
Source: BMC Health Serv Res. 2019 May 3;19:284. doi: 10.1186/s12913-019-4112-3 (PMC6499968; doi:10.1186/s12913-019-4112-3)
Supplement: Supplementary file 1 — Scenario and medical record of Patient 1 (John Thompson). Scenario and medical record of Patient 1 (John Thompson) (DOCX 14 kb) [file 12913_2019_4112_MOESM1_ESM.docx]

**Additional File 1: Scenario and simulated medical record of Patient 1 (John Thompson)**

This patient has had T2DM for five years, a BMI of 29 and is on triple therapy of metformin, sulfonylurea and a DPP4 inhibitor at maximal tolerated doses. He has a history of depression for which he has been hospitalised on two occasions. He has urinary symptoms on close questioning that are more genitourinary than osmotic in origin. He has developed early maculopathy. He has had HbA1c levels that have ranged between 53 and 58 mmol/mol for the last two years. He is very keen on achieving better glycaemic control and has heard that insulin is much the best treatment for T2DM in the long run.

**Simulated medical record Patient 1 (John Thompson)**

**Name:** THOMPSON, John

**Date of birth:** 01-Jan-1966 (52 years old)

**Problems**

***Active***

23-Jan-2018 Background diabetic retinopathy. Review routine retinal screening

09-Jun-2015 Chronic depression

14-Apr-2015 Essential hypertension

27-Feb-2015 [X] Intentional self-harm. Hospitalised

24-Dec-2014 [X] Severe depressive episode Hospitalised

without psychotic symptoms

14-Aug-2013 Type 2 diabetes mellitus

**Medication**

***Acute***

***Repeat***

| Sertraline 100mg tablets | One to be taken each day | 28 tablet |
| --- | --- | --- |
| Atorvastatin 20 mg tablets | One to be taken each night | 28 tablet |
| Enalapril 5mg tablets | One to be taken twice a day | 56 tablet |
| Gliclazide 80mg tablets | Two to be taken twice a day | 112 tablet |
| Metformin 500mg tablets | Two to be taken twice a day | 112 tablet |

**Adverse reactions / intolerances**

None recorded

**Health status**

11-Jun-2018 O/E - blood pressure reading 128/78 mmHg

23-Jan-2018 Ex-light smoker 1-9 /day

23-Jan-2018 Alcohol consumption 4 U/week

23-Jan-2018 Body mass index 29 kg/m2

23-Jan-2018 O/E - weight 94 kg

23-Jan-2018 O/E - height 180 cm

**Planned events**

11-Jun-2018 ACEi or ARB monitoring advised

11-Jun-2018 No PHQ9 recorded

11-Jun-2018 Offer Diabetes UK Information Prescription

11-Jun-2018 No record of initial alcohol screening

**Consultations**

11-Jun-2018 Entered via nurse

Result O/E - left foot pulses present • O/E - right foot pulses present • 10g monofilament sensation L foot normal • 10g monofilament sensation R foot normal • O/E - Vibration sense of left foot normal • Vibration sense of right foot normal • O/E - Left diabetic foot at low risk • O/E - Right diabetic foot at low risk

11-Jun-2018 Entered via GP

Problem **Type 2 diabetes mellitus** *(Review)*

History Background retinopathy maculopathy

Examination O/E - blood pressure reading 128/78 mmHg • Haemoglobin A1c level - IFCC standardised 58 mmol/mol • eGFR >60 • Urine albumin:creatinine ratio 1.4 mg/mmol • Serum cholesterol 4.4 mmol/L

Comment Refer Eye Unit

23-Jan-2018 Entered via administrator

Examination O/E - height 180 cm • O/E - weight 94 kg • Body mass index 29.1 kg/m2 • Ideal weight 74.5 kg

Social Alcohol consumption 4 U/week • Ex smoker •Stopped smoking (12-Dec-2017) • Ex-light smoker (1-9/day)

Additional Takes inadequate exercise

23-Jan-2018 Entered via nurse

Result O/E - left foot pulses present • O/E - right foot pulses present • 10g monofilament sensation L foot normal • 10g monofilament sensation R foot normal • O/E - Vibration sense of left foot normal • Vibration sense of right foot normal • O/E - Left diabetic foot at low risk • O/E - Right diabetic foot at low risk

23-Jan-2018 Entered via nurse

Examination O/E - blood pressure reading 126/80 mmHg • Haemoglobin A1c level - IFCC standardised 53 mmol/mol • Serum creatinine >60 umol/L • Urine albumin:creatinine ratio 0.8 mg/mmol

23-Jan-2018 Entered via GP

Problem **Type 2 diabetes mellitus** *(Review)*

Examination Serum cholesterol 5.4 mmol/L

Comment No change to current therapy. No hypos. Switch simvastatin to atorvastatin 20mg nocte. Review 6/12

24-Jul-2017 Entered via GP

Problem **Type 2 diabetes mellitus** *(Review)*

Examination Haemoglobin A1c level - IFCC standardised 55 mmol/mol

Comment Slightly anxious during consultation. Seeing CPN next week. Review diabetic control 6/12.

**Values and Investigations**

11-Jun-2018 Serum cholesterol 4.4 mmol/L

11-Jun-2018 Urine albumin:creatinine ratio 1.4 mg/mmol

11-Jun-2018 eGFR >60 umol/L

11-Jun-2018 Haemoglobin A1c level - IFCC standardised 58 mmol/mol

11-Jun-2018 O/E - blood pressure reading 128/78 mmHg

23-Jan-2018 Alcohol consumption 4 U/week

23-Jan-2018 Ideal weight 74.5 kg

23-Jan-2018 Body mass index 29 kg/m2

23-Jan-2018 O/E - weight 94 kg

23-Jan-2018 O/E - height 180 cm

23-Jan-2018 Urine albumin:creatinine ratio 0.8 mg/mmol

23-Jan-2018 eGFR >60 umol/L

23-Jan-2018 Haemoglobin A1c level - IFCC standardised 53 mmol/mol

23-Jan-2018 O/E - blood pressure reading 126/80 mmHg

23-Jan-2018 Serum cholesterol 5.4 mmol/L

24-Jul-2017 Haemoglobin A1c level - IFCC standardised 55 mmol/mol
