# Supplementary material for: Intensification to injectable therapy in type 2 diabetes: mixed methods study (protocol)
Source: BMC Health Serv Res. 2019 May 3;19:284. doi: 10.1186/s12913-019-4112-3 (PMC6499968; doi:10.1186/s12913-019-4112-3)
Supplement: Supplementary file 2 — Scenario and medical record of Patient 2 (Jane Smith). Scenario and medical record of Patient 2 (Jane Smith) (DOCX 14 kb) [file 12913_2019_4112_MOESM2_ESM.docx]

**Additional File 2: Scenario and simulated medical record of Patient 2 (Jane Smith)**

This patient has had T2DM for four years. She has a BMI of 28 and HbA1c of 64 mmol/mol. She could not tolerate more than a small dose on metformin initially and had to stop this eventually because of persistent and troublesome GI side effects. Over the years, she has tried several different classes of oral glucose lowering therapies and experienced significant intolerance with all except DPP4 inhibitors and sulphonylureas. She is currently taking a DPP4 and a sulphonylurea. She noticed that her clothes seem looser of late.

**Simulated medical record of Patient 2 (Jane Smith)**

**Name:** SMITH, Jane (Mrs)

**Date of birth:** 11-Jun-1960 (58 years old)

**Problems**

***Active***

11-Jun-2018 Adverse reaction to Pioglitazone Hydrochloride

05-Feb-2014 Type 2 diabetes mellitus

12-Sep-1985 Irritable bowel syndrome

**Current Medication**

***Repeat***

| Mebeverine 135 mg tablets | One to be taken three times a day twenty minutes before food | 100 tablet |
| --- | --- | --- |
| Simvastatin 20 mg tablets | One to be taken at night | 28 tablet |
| Enalapril 5mg tablets | One to be taken each day | 29 tablet |
| Gliclazide 80mg tablets | Two to be taken twice a day | 112 tablet |
| Alogliptin 25mg tablets | One to be taken daily | 28 tablet |

**Previous Medication**

| Empagliflozin 10mg | One to be taken each day | 28 tablet |
| --- | --- | --- |
| Metformin 500mg tablets | Two to be taken twice a day | 112 tablet |

**Adverse reactions / intolerances**

11-Jun-2018 Adverse reaction to Empagliflozin

11-Jun-2018 Adverse reaction to Pioglitazone Hydrochloride

11-Jun-2018 Metformin not tolerated

**Health status**

11-Jun-2018 Cervical smear: negative

11-Jun-2018 Alcohol consumption 6 U/week

11-Jun-2018 O/E - blood pressure reading 132/80 mmHg

11-Jun-2018 Body mass index 28.1 kg/m2

11-Jun-2018 O/E - weight 86 kg

11-Jun-2018 O/E - height 175 cm

11-Jun-2018 Cigarette smoker 10 /day

**Planned events**

11-Jun-2018 ACEi or ARB monitoring advised

11-Jun-2018 No PHQ9 recorded

11-Jun-2018 Offer Diabetes UK Information Prescription

11-Jun-2018 No record of initial alcohol screening

**Consultations**

11-Jun-2018 Entered via administrator

Examination O/E - height 175 cm • O/E - weight 86 kg • Body mass index 28.1 kg/m2 • Ideal weight 70.4 kg • O/E - blood pressure reading 132/80 mmHg

Procedure Cervical neoplasia screen GMS: GMS

Result Cervical smear: negative

Social Alcohol consumption 6 U/week

Cigarette smoker 10/day

Additional Takes inadequate exercise

11-Jun-2018 Entered via nurse

Result O/E - left foot pulses present • O/E - right foot pulses present • 10g monofilament sensation L foot normal • 10g monofilament sensation R foot normal • O/E - Vibration sense of left foot normal • Vibration sense of right foot normal • O/E - Left diabetic foot at low risk • O/E - Right diabetic foot at low risk

16-Jan-2018 Entered via administrator

Examination O/E - height 175 cm • O/E - weight 89 kg • Body mass index 29.1 kg/m2 • Ideal weight 70.4 kg • O/E - blood pressure reading 126/78mmHg

16-Jan-2018 Entered via nurse

Result O/E - left foot pulses present • O/E - right foot pulses present • 10g monofilament sensation L foot normal • 10g monofilament sensation R foot normal • O/E - Vibration sense of left foot normal • Vibration sense of right foot normal • O/E - Left diabetic foot at low risk • O/E - Right diabetic foot at low risk

16-Jan-2018 Entered via GP

Problem **Type 2 diabetes mellitus** *(Review)*

Examination Haemoglobin A1c level - IFCC standardised 64 mmol/mol

Comment Increase gliclazide to 160mg bd. Review in 6/12

17-Jul-2017 Entered via GP

Problem **Type 2 diabetes mellitus** *(Review)*

Examination Haemoglobin A1c level - IFCC standardised 54 mmol/mol

Comment Empagliflozin caused thrush. To stop. Add alogliptin 25mg od

Watch gliclazide 80mg bd

05-Jan-2017 Entered via GP

Problem **Type 2 diabetes mellitus** *(Review)*

Examination Haemoglobin A1c level - IFCC standardised 62 mmol/mol

Comment Add empagliflozin 10mg and watch gliclazide. Review in 6/12

12-Jul-2016 Entered via GP

Problem **Type 2 diabetes mellitus** *(Review)*

Examination Haemoglobin A1c level - IFCC standardised 68 mmol/mol

Comment Metfomin caused GI side-effects (diarrhoea). To stop. Add gliclazide 80mg bd. Review in 6/12

**Values and Investigations**

11-Jun-2018 Cervical smear: negative

11-Jun-2018 Alcohol consumption 6 U/week

11-Jun-2018 O/E - blood pressure reading 132/80 mmHg

11-Jun-2018 Ideal weight 70.4 kg

11-Jun-2018 Body mass index 28.1 kg/m2

11-Jun-2018 O/E - weight 86 kg

11-Jun-2018 O/E - height 175 cm

11-Jun-2018 Cigarette smoker 10 /day

11-Jun-2018 Body mass index 28 kg/m2

17-May-2018 Serum cholesterol 4.6 mmol/L

17-May-2018 Urine albumin:creatinine ratio 0.9 mg/mmol

17-May-2018 eGFR >60 umol/L

17-May-2018 Haemoglobin A1c level - IFCC standardised 64 mmol/mol

16-Jan-2018 O/E - blood pressure reading 126/78 mmHg

16-Jan-2018 Ideal weight 70.4 kg

16-Jan-2018 Body mass index 29.1 kg/m2

16-Jan-2018 O/E - weight 89 kg

16-Jan-2018 O/E - height 175 cm

16-Jan-2018 Haemoglobin A1c level - IFCC standardised 64 mmol/mol

16-Jan-2018 Serum cholesterol 4.3 mmol/L

16-Jan-2018 Urine albumin:creatinine ratio 1.1 mg/mmol

16-Jan-2018 eGFR >60 umol/L

17-Jul-2017 Haemoglobin A1c level - IFCC standardised 54 mmol/mol
