# Supplementary material for: Intensification to injectable therapy in type 2 diabetes: mixed methods study (protocol)
Source: BMC Health Serv Res. 2019 May 3;19:284. doi: 10.1186/s12913-019-4112-3 (PMC6499968; doi:10.1186/s12913-019-4112-3)
Supplement: Supplementary file 3 — Scenario and medical record of Patient 3 (Gary Jones). Scenario and medical record of Patient 3 (Gary Jones) (DOCX 14 kb) [file 12913_2019_4112_MOESM3_ESM.docx]

**Additional file 3: Scenario and simulated medical record of Patient 3 (Gary Jones)**

This patient will be a bus driver with a six year history of T2DM. He also has hypertension and hypercholesterolaemia. He has a BMI of 36. His current glucose lowering medication includes metformin, sitagliptin and empagliflozin at maximum recommended doses. He refused to take pioglitazone in case of further weight gain. His HbA1c is 68 mmol/mol. He wants improved glycaemic control as he has now developed early diabetic retinopathy. Whilst he is worried about his elevated HbA1c, he does not want to risk hypoglycaemia. His driving medical is due in 3 months’ time.

**Simulated medical record of Patient 3 (Gary Jones)**

**Name:** JONES, Gary

**Date of birth:** 14-Feb-1964 (54 years old)

**Problems**

***Active***

11-Jun-2018 Background diabetic retinopathy. Check in 6/12

11-Jul-2012 Type 2 diabetes mellitus

12-Jun-2008 Hypercholesterolaemia

08-April-2008 Essential hypertension

**Current Medication**

***Acute***

| Empagliflozin 25mg tablets | One to be taken each day | 28 tablet |
| --- | --- | --- |
| Amlodipine 5 mg tablets | One to be taken each night | 28 tablet |

***Repeat***

| Atorvastatin 40 mg tablets | One to be taken each night | 28 tablet |
| --- | --- | --- |
| Indapamide 1.5mg modified-release tablets | One to be taken each morning | 30 tablet |
| Enalapril 20mg tablets | One to be taken each day | 28 tablet |
| Sitagliptin 100mg tablets | One to be taken each day | 28 tablets |
| Metformin 500mg tablets | Two to be taken twice a day | 112 tablet |

**Previous Medication**

| Empagliflozin 10mg tablets | One to be taken each day | 28 tablet |
| --- | --- | --- |

**Adverse reactions / intolerances**

None recorded

**Health status**

11-Jun-2018 O/E - blood pressure reading 140/84 mmHg

11-Jun-2018 Body mass index 36.2 kg/m2

11-Jun-2018 O/E - weight 111 kg

11-Jun-2018 O/E - height 175 cm

09-Jan-2018 Cigarette smoker 20 /day

09-Jan-2018 Alcohol consumption 4 U/week

**Planned events**

11-Jun-2018 ACEi or ARB monitoring advised

11-Jun-2018 No PHQ9 recorded

11-Jun-2018 Offer Diabetes UK Information Prescription

11-Jun-2018 No record of initial alcohol screening

**Consultations**

11-Jun-2018 Entered via GP

Examination Haemoglobin A1c level - IFCC standardised 68 mmol/mol • eGFR >60 umol/L • Urine albumin:creatinine ratio 1.3 mg/mmol • Serum cholesterol 5.8 mmol/L

11-Jun-2018 Entered via nurse

Result O/E - left foot pulses present • O/E - right foot pulses present • 10g monofilament sensation L foot normal • 10g monofilament sensation R foot normal • O/E - Vibration sense of left foot normal • Vibration sense of right foot normal • O/E - Left diabetic foot at low risk • O/E - Right diabetic foot at low risk

11-Jun-2018 Entered via administrator

Examination O/E - height 175 cm • O/E - weight 111 kg • Body mass index 36.2 kg/m2 • Ideal weight 70.4 kg • O/E - blood pressure reading 140/84 mmHg

Comment Recheck bp in 1/12 and ? increase enalapril

09-Jan-2018 Entered via administrator

Examination O/E - height 175 cm • O/E - weight 107 kg • Body mass index 34.9 kg/m2 • Ideal weight 70.4 kg

Social Alcohol consumption 4 U/week • Cigarette smoker 20/day

Additional Takes inadequate exercise

09-Jan-2018 Entered via nurse

Examination O/E - blood pressure reading 146/94 mmHg • Haemoglobin A1c level - IFCC standardised 64 mmol/mol • eGFR >60 umol/L • Urine albumin:creatinine ratio 1.1 mg/mmol • Serum cholesterol 5.0 mmol/L

Medication Amlodipine 5mg tablets One to be taken each day, 28 tablet

Comment Increase antihypertensive agents

Increase empagliflozin to 25mg od

Add amlodipine 5mg od

Result O/E - left foot pulses present • O/E - right foot pulses present • 10g monofilament sensation L foot normal • 10g monofilament sensation R foot normal • O/E - Vibration sense of left foot normal • Vibration sense of right foot normal • O/E - Left diabetic foot at low risk • O/E - Right diabetic foot at low risk

10-Jul-2017 Entered via GP

Problem **Type 2 diabetes mellitus** *(First)* (11-Jul-2012)

Medication Empagliflozin 10mg tablets take one daily, 28 tablet

Comment Weight has increased. HbA1c is now 66. Does not want pioglitazone. Reluctant re injectable therapy. Add empagliflozin 10mg od

**Values and Investigations**

11-Jun-2018 Serum cholesterol 5.8 mmol/L

11-Jun-2018 Urine albumin:creatinine ratio 1.3 mg/mmol

11-Jun-2018 eGFR >60 umol/L

11-Jun-2018 Haemoglobin A1c level - IFCC standardised 68 mmol/mol

11-Jun-2018 O/E - blood pressure reading 140/84 mmHg

11-Jun-2018 Ideal weight 70.4 kg

11-Jun-2018 Body mass index 36.2 kg/m2

11-Jun-2018 O/E - weight 111 kg

11-Jun-2018 O/E - height 175 cm

09-Jan-2018 Cigarette smoker 20 /day

09-Jan-2018 Alcohol consumption 4 U/week

09-Jan-2018 Ideal weight 70.4 kg

09-Jan-2018 Body mass index 34.9 kg/m2

09-Jan-2018 O/E - weight 107 kg

09-Jan-2018 O/E - height 175 cm

09-Jan-2018 Serum cholesterol 5 mmol/L

09-Jan-2018 Urine albumin:creatinine ratio 1.1 mg/mmol

09-Jan-2018 eGFR >60 umol/L

09-Jan-2018 Haemoglobin A1c level - IFCC standardised 64 mmol/mol

09-Jan-2018 O/E - blood pressure reading 146/84 mmHg
